# Students, Participatory Design, and Serious Games in a Response to: ‘No Algorithmization without Representation: Pilot Study on Regulatory Experiments in an Exploratory Sandbox’

**DOI:** 10.1007/s44206-022-00024-0

**Published:** 2022-10-25

**Authors:** Brian Ballsun-Stanton

**Affiliations:** Macquarie University, Sydney, Australia

**Keywords:** Philosophy, Open Data, Serious Games, COVID-19 contact tracing apps, Blockchain

## Abstract

No Algorithmization without Representation tracked a cohort of ‘Lithopy’ crypto-government sandbox participants in a longitudinal study looking at COVID-19 contact tracing app acceptance. These survey responses extended experiences with theoretical blockchain town governance by also tracking reasons for and against compliance with contact tracing apps. They found that the expressed opinions of students were incoherent and demanded technical or policy responses outside of the students’ direct experiences. In this response to that paper, I leverage the paper’s (commendable) open data to suggest that the sandbox’s claims of ‘No Algorithmization without Representation’ is a rediscovery of participatory design within the context of the serious games movement. While Role-Playing Games and War Games are excellent pedagogic tools xor planning tools—using undergraduate students’ participation in them as the basis of a claim for increased representation in technology policy is a bold claim. This claim is not fully substantiated by the paper’s data. Nevertheless, there is a need for better decision-making and public representation within technology design and policy-making spaces—making the claim for serious games as a meaningful public policy contribution not without merit.

The authors posit a regulatory experimental philosophy sandbox as a function of prompting intuitions about digital territoriality/representation. They assert that these sandboxes provide a mechanism for democratic participation in technological regulation. However, the data from the blockchain ‘sandbox’ and installation decisions around the COVID-19 contact tracing app do not support the bold claims around sandboxes being a far better participation mechanism for app governance as a viable alternative to ‘the reductionist views of regulation of algorithmic governance involving only ethical or technical issues.’ However, the longitudinal insights gathered as part of this experimental philosophy study are worth discussing and there are useful routes forward towards applying participatory design and simulations as serious games towards the norms, policies, and designs of online systems.

Before all else, however, the authors must be specifically and fully commended: they published their data and their analysis code such that I could explore their data outside the context of their paper. As part of a burgeoning tradition in philosophy this is to be celebrated! Being able to look at their code and their data allowed me to structure my response to their work, understand their research through a different frame, and explore their conclusions. This level of data publication is the minimum standard we should hold other works to—even when the works are explicitly hypothesis-generating rather than hypothesis-testing (Ross & Ballsun-Stanton, 2022).

This review will explore two topics from the data in ‘No Algorithmization without Representation: Regulatory Experiments in Exploratory Sandbox’:

1. What can we learn from the incoherence of responses in the surveys over time?
2. Is a sandbox, inviting participants to reflect and engage with a scenario, a useful experimental philosophy tool?

I fully agree with the thesis of the paper, arguing that more participatory design can result in better and more democratic outcomes from technology and policy (Muller & Kuhn, 1993; Schuler & Namioka, 1993; Hartman et al., 2010; Lodato & DiSalvo, 2018; Blomkamp, 2018). Unfortunately, my response reflects a different reading of the survey results and a different framing of the paper’s conclusions. This paper, rather than using its data to present arguments for democratic participation in technological decision-making, demonstrates how respondents apply pre-extant epistemological frames in the presence of uncertainty. While I agree with the thesis of the paper and the use of simulations and sandboxes to support policy-making, I do not believe that the survey results presented are useful evidence for the paper’s thesis.

## Unpacking Response Incoherence

The researchers say:

Based on the Lithopy experience, we claim that exploratory sandboxes democratize the decision-making processes and support negotiations of diverse groups about concrete scenarios ... It is a training and testing ground for understanding the issues of power, stakes and ownership when facing a new code or missing regulations.

They then validate the ‘blockchain regulatory sandbox’ by eliciting responses from prior participants who have opinions about COVID-19 contact tracing apps. They assert a commonality of purpose in these disparate domains by focusing on the centrality of governmental surveillance, allowing a conflation of public health responses with quasi-libertarian government-by-technological-contract scenarios. In practice, therefore, this longitudinal experiment measures trust-in-government through a proxy of technological objections. While the authors’ claim:

With this pilot study, we mapped the different reasons for accepting or rejecting algorithmic services and the different levels of trust in the oversight. We interpret them as something that changes depending on the experiences with agency, participation and representation in the design and policy process.

Looking at the data at: https://zenodo.org/record/5949422 and https://public.tableau.com/app/profile/denisa.kera/viz/BookLithopy/FutureofRegTechHowtoRegulateAlgorithms we can see a great deal of epistemic uncertainty in respondents full-text responses: ‘I would like to know more about this, because I am not really sure about the pros and cons but a the moment I don’t think so.’ (lithopy) and the common shorthand of ‘data leak’ in the e-rouska app responses suggests that in both cases, survey designers have fallen afoul of the XY Problem:

The XY problem is asking about your attempted solution rather than your actual problem. This leads to enormous amounts of wasted time and energy, both on the part of people asking for help, and on the part of those providing help (Stack Exchange Meta Community, 2010).

While the XY problem is a severe issue on question and answer sites like Stack Overflow, where querents leap immediately to questions about implementing their first imagined solution; the sandbox and surveys jumped immediately to questions about possible trust-solutions rather than establishing a solid epistemological baseline. We do not know if the university students responding to these surveys had any detailed understanding of how the apps operated, what data was collected, or how it was shared. Instead, there are indications that the respondents used the app as a proxy for trust in government/institutions/companies/technology. Thus, when the authors asked participants about possible solutions to application adoption issues, the answers could be viewed as effectively random, reflecting a distorted reflection in the respondents’ trust-in-government and/or ideological stance towards COVID-19:

The group that installed the app seems to express more resignation, and perhaps [scepticism], toward technical interventions in April 2021, while the rejectors seem more willing to demand the technical but also institutional interventions.

There was no evidence that the sandbox promoted engagement or increased understanding within the context of lithopy. Instead, it seems as if the participants prior epistemic frames were used when they were uncertain about the specific issues surrounding a problem. While this might be viewed as a survey-design issue, I prefer to think of this response incoherence correctly picking up the epistemic incoherence of the participants’ policy preferences, trust in government, and technological understanding.

In the lithopy sandbox survey results, this trend towards prior frames is even more pronounced:

When we look at the [visualisation] on Image 3 from Tableau story slide 1, a typical supporter of algorithmic rule is someone with no knowledge of blockchain technology but awareness of governance and regulation issues (64 %). ... The strongest opponents of algorithmic services (Image 5) remained the self-assessed experts in cryptocurrency with no knowledge of governance and regulatory issues..

When respondents with domain expertise prefer the solution which resides outside of their individual domain expertise in both directions, the questions being asked to them are premature. Therefore, I don’t read the responses as a demand for democratic or even representative-based representation. Instead, the responses are proxies for trust-in-government or knowledge-of-system. We should consider these responses as affirmations of individual ideologies absent specific expertise and understanding of the problems at hand.

## On the Uses of Sandboxes

I am a long-time player of tabletop Role-Playing games and have engaged in multiple Live-Action Role-Playing games over two decades participating in and publishing in the hobby^1^. These games maintain a long tradition with more operationally focused political simulations and military war-games that militaries throughout the world have found useful both for training and for tactical, operational, and strategic modelling (Mc Grady & Trentacoste, 2014). In my personal experience, these two genres blended as part of the National Security Decision Making Game (NSDMG) series run in the early 2000s at conventions around the United States. In these games, I pretended to be a member of the leadership of various countries, trying to negotiate and make policy in collaboration with and in opposition to my fellow players. As an undergraduate, I found these games to be an enormously useful learning experience in leadership, geopolitics, and ‘lived’ country-specific concerns presented in a novel and engaging format. Looking at the PAXsims blog and what my colleagues down the hall in the Security Studies and Criminology department at Macquarie University are doing—the tradition of geopolitical simulations qua wargames, these ‘Serious Games,’ remain an effective teaching and research tool (Mayer, 2009; Smith & Johnson, 2020; Djaouti et al., 2011; Lim & Jung, 2013).

At no point, while participating in these games as an undergraduate, did I believe that I was making effective contributions to national policy. They were a fun and engaging experience that improved my understanding of the world. I did not, however, view these games as an element of democratic participatory design. Serious games can certainly be run for education or for policy-makers, for military staff, or even as a way of imagining possible futures and policies in community or corporate governance. It is seldom wise to combine these two purposes in one single session.

Within the paper, the authors assert a similar learning experience to the one I experienced:

The exploratory sandbox helped the participants to envision a more active role in the design and regulation of the new services, which we believe influenced their final responses and preference for independent oversight. By experiencing hybrid and iterative engagements that crossed the divisions between the code and values, new infrastructures and old institutions, the participants realized their limits on every level.

And if the paper’s questions spoke to a participant’s personal understanding-of-scenario, or looked at attitude change pre- and post- workshop, this would be an effective look at how serious games could educate within an technological application policy-setting context.

Unfortunately, the authors did not take this approach. They claim:

To summarize the experiences and feedback from the sandbox together with the later responses on contact tracing, we are using the credo of the American colonists demanding ’no taxation without representation’ and claim that the algo-colonists are demanding ’no algorithmization without representation’. The new data or algo-subjects under surveillance in the new (digital) territories are trying to balance the different forces, opportunities, and threats.

As sympathetic as I am to these demands, they are simply not supported by the responses. A desire for oversight coupled with low social trust and a question prompt for independent audits is not a clear call for participatory design, much less ‘representation in algorithmization.’ While one free text response in 4.c said: ‘with limited transparency, open source and owned by the city so it enables the citizen to see the issues and improve the platform’—there seem to be no coherent themes around an understanding of what ‘open source’ means in to participants in this sandbox’s context or expressed understanding of nuances around public/private ownership.

Using a sandbox or Role-Glaying Game as a pedagogical tool around promoting or engaging in participatory design is an excellent idea. Using a sandbox as a pedagogical and policy-making tool simultaneously is terrifying. The authors claim:

The experimental governance of emerging services in the sandboxes offers a pragmatic alternative to the hierarchical, command-and-control models of governance, but also to the aspirational ethical frameworks and recommendations that come too late or achieve too little.

Their claim echoes with Stafford Beer’s ‘Viable Systems Model’ and the various compromises in its attempted implementation in Chile (Medina, 2011). In Beer’s model, constituent participation through feedback loops is fundamental to a functioning system. And it seems that this sandbox and the desires of participants inside the sandbox, echo that desire for an effective feedback loop during design and implementation.

However, from the perspective of the authors’ claim that: ‘The exploratory sandbox allows the participants to experience the alternative futures and make more informed decisions or negotiate the preferred interventions,’ I must ask: ‘Who bells the cat?’ In my experience, initial conditions strongly determine the course and experience of any simulation or Role-Playing game. The rather conservative post-Clinton era Department of Defence veterans running the NSDMG had very firm opinions on the way economies and societies worked and those opinions played out in game. As an undergraduate experiencing a simulated reality of geopolitics for the first time, I was unprepared to discuss, much less debate, the theoretical or factual assumptions of the scenario.

While this paper’s claim towards a viable system model/participatory democracy is praiseworthy, as ‘Instead of a strong and empirically tested hypothesis, the pilot study shows the need for more experimental and longitudinal approaches identifying the regulatory expectations and experiences of the citizens,’ neither the paper’s data nor my own experiences in playing and running simulations support this as a better option. It is, of course, preferable to engage and involve all involved users *nee* citizens in algorithmic changes. The authors’ claims that this, ‘hybrid and experimental approach to algorithmic services is a viable alternative to the reductionist views of regulation of algorithmic governance involving only ethical or technical issues,’ are not substantiated by Chile’s response to Beer’s models, the lack of overwhelming dominance of war-games in military planning to other forms of military planning, or my own experiences in attending serious games. A sandbox as a means of encouraging participatory democracy and as a venue for a Serious Game is excellent—but if it alone was the decision-making modality, the designers of the initial conditions of the sandbox would have most of the power.

## A Response to the Paper’s Thesis

The authors submit the idea of sandboxes as trading zones which:

helped the participants to envision a more active role in the design and regulation of the new services, which we believe influenced their final responses and preference for independent oversight. By experiencing hybrid and iterative engagements that crossed the divisions between the code and values, new infrastructures and old institutions, the participants realized their limits on every level.

A sandbox as they describe where normal users can play with a technology, and thereby make claims around representation and governance is a good, if incomplete, idea. Instead, if we think of one of Galison’s Trading Zones where experimentalists create temporary languages with instrumentalists and theoreticians, we can map equivalent trading zones to the stakeholders in one of these sandboxes (Galison, 1997). The stakeholders can develop working trading zones as they ‘play’ with policy-controls for the technologies on offer. While a sandbox may not be able to overcome Mazur’s ‘Rashomon effect’ through the creation of local languages with a single iteration, using an iterated serious game/wargame setup for policy experimentation, experimental philosophy, and community building is not outside the realm of imagination (Mazur, 1998).

I, however, do not believe the paper’s data supports the author’s thesis that representation and non-governmental industrial oversight would create better and more positive outcomes for decentralized governance or government-adjacent technologies. It is clear from the survey responses that, despite participating in the sandbox, users did not have appropriate mental models of the technologies in question. Instead, users mapped their preexisting ideological approaches onto technological questions and showed an exceptional unwillingness to explore the governance technologies. However, this paper is a worthwhile contribution to discussions of the philosophy of technology as it contributes to the conversation around how university students model risks and benefits in technologies that they do not understand. The authors have published their code and their data and have created an excellent basis for future discussion—even if their central claim is not supported by their data.

Who could possibly represent this group of uncurious and ideologically driven users to stakeholders? How would the representation occur? If we were to respect the group’s wishes and remove the government from the equation, who would be able to enforce oversight inside the cabals, scams, and hype-driven ignorance of decentralised-ledger based approaches to technology, public policy, or direct governance (see Apenwarr, 2021; Gault, 2021; Graham, 2021)? How would they, after suffering intentional or unintentional losses to a difficult-to-understand system predicated on the irreversibility of transactions, find justice or mitigate harms? A sandbox which can improve the intuitive models of users is a far more possible and pragmatic thing than a sandbox which can enable effective user representation to these lawless stakeholders.

If this paper could be the start of a ‘publish your code!’ trend in the experimental philosophy, philosophy of information, or the philosophy of technology, the paper will have been of benefit. If we can use the responses from students to explore better approaches for gaming out risks and technological understandings in crises, this paper will have been of benefit. If an activist can use this paper and this discussion to argue for proper application testing and involvement of users during development of governmental policy and apps, this paper will have been of benefit. Finally, this paper serves as a useful proof of concept around scenario based experiments in experimental philosophy—offering some data, lessons learned, and opportunities for further research between serious games, participatory design, and philosophy.

## Data Availability

The datasets generated during and/or analysed during the current study are available in the original paper’s Zenodo repository, https://zenodo.org/record/5949422 and within the original paper’s Tableau dashboard https://public.tableau.com/app/profile/denisa.kera/viz/BookLithopy/FutureofRegTechHowtoRegulateAlgorithms.

## Footnotes

1 See my participation and moderation of the Role-Playing Games Stack Exchange for more details.
